# Mature tertiary lymphoid structure associated CD103+ CD8+ Trm cells determined improved anti-tumor immune in breast cancer

**DOI:** 10.3389/fonc.2025.1480461

**Published:** 2025-01-24

**Authors:** Qin Fang, Shuru Chen, Xiaoyue Chen, Wei Zou, Di Chen, Yukang Huang, Chucheng Wu

**Affiliations:** Huizhou Central People’s Hospital, Huizhou, China

**Keywords:** breast cancer, tertiary lymphoid structures, CD103+ CD8+ Trm cells, prognosis factors, tumor immunology

## Abstract

**Background:**

Although tertiary lymphoid structures (TLS) play crucial roles in the anti-tumor immune response and are associated with favorable prognoses in many solid tumors, the precise mechanisms by which TLSs enhance anti-tumor immunity remain poorly understood. The current study aimed to explore the relationship between the maturity of tertiary lymphoid structures and their key immune cells in combating breast cancer.

**Patients and methods:**

In this study, we utilized immunofluorescence and H&E staining to detect tumor-resident memory T cells (Trm) and assess the maturity of TLS, analyzing their distribution and proportion in an annotated cohort of 95 breast cancer patients.

**Results:**

The presence of tumor-associated TLSs was correlated with an improved prognosis in patients with breast cancer. The proportion of CD8+CD103+ resident memory T cells and natural killer (NK) cells within the TLSs was significantly higher than that in areas outside of these structures. Additionally, the proportions of CD103+ CD8+ Trm cells and NK cells were significantly increased with the gradual maturation of TLS. Furthermore, the secretion function of effector molecules by CD8+ CD103+ Trm cells and NK cells within TLSs was significantly enhanced, indicating a strong correlation between the effector function of CD103+ CD8+ Trm and NK cells and the maturity of TLSs.

**Conclusion:**

Our study identifies potential additional prognostic information for the clinical prognosis of breast cancer patients, underscoring the prognostic significance of immune cells within TLS, with a particular focus on CD103+ CD8+ Trm cells and NK cells.

## Background

Breast cancer is the most frequently diagnosed cancer in women and affects a large percentage of women (1). Although significant advancements have been made in the early detection and treatment of breast cancer, it continues to be the second leading cause of cancer-related deaths in women, following lung cancer (2). While breast cancer is generally considered less immunogenic than cancers like melanoma or lung cancer, it remains among the top ten most immune-infiltrated tumors according to Mandal et al (3). This highlights that, despite its comparatively lower immunogenicity, immune infiltration plays a significant role in the breast cancer microenvironment. Nonetheless, recent studies have consistently demonstrated that the tumor microenvironment of breast cancer encompasses a varied array of cell populations from both the innate and adaptive immune systems. These populations have proven to be of biological and clinical significance to different extents (4). To identify effective therapeutic strategies against BC progression, it is necessary to provide a more profound elucidation of the histological features.

Over the past few decades, cancer immunotherapy has transformed from a promising treatment approach into a substantial clinical reality (37828275). Traditional treatments like surgery, chemotherapy, and radiotherapy primarily target the tumor cells directly, often overlooking the critical roles played by the TME and the immune system in cancer progression and response to treatment. The TME consists of various components, including immune cells, fibroblasts, endothelial cells, extracellular matrix, and signaling molecules, all of which can influence tumor growth, metastasis, and resistance to therapy (5, 6). Recent studies and clinical trials, especially those involving immunotherapy, have underscored the importance of considering the immune contexture in cancer treatment. Immunotherapy, such as immune checkpoint inhibitors (ICIs), has demonstrated significant success by targeting the interactions between tumor cells and immune cells, thereby reactivating the immune system to recognize and attack cancer cells. This success highlights the potential of therapies that modulate the immune environment to enhance cancer treatment efficacy (7, 8).

Ectopic accumulations of immune cells including B and T cell is initially identified as tertiary lymphoid structures (TLSs) that develops in the context of chronic inflammation or cancer (9). TLSs often exhibit features of conventional secondary lymphoid organs, including high endothelial-like vessels that express peripheral node addressin (PNAd), a T-cell zone with mature DCs, and adjacent B-cell zone with follicular DCs and B cells (9, 10). The presence of TLSs is documented in association with control of cancer growth and better prognosis in a wide variety of primary and metastatic solid tumors of human (10, 11). To date, how BC-associated TLSs augment anti-tumor immunity remains incompletely understood.

In this study, we reported the finding that tissue-resident memory CD8+ Trm cells within tumor-associated TLS correlate with improved prognosis in patients with breast cancer. We identified that CD103+ CD8+ Trm cells within tumor-associated TLS contributed to anti-tumor immune response in breast cancer. To sum up, this study further revealed relationship between CD103+ CD8+Trm cells, NK cells and TLS, shedding light on the role of CD103+ CD8+Trm cells and NK cells within TLS in promoting cancer immunotherapy.

## Materials and method

This study is a retrospective study (approval number: GASTO-22-01-007, November 2022). An annotated cohort of 95 untreated primary invasive breast carcinoma and ductal breast carcinoma, collected from female patients diagnosed and treated at Huizhou Centers Hospital between 2017 and 2018, was analyzed. Oral informed consent was obtained from the patients. A pathologist specializing in breast cancer reviewed the clinical and pathological features of the patients involved in this study. The experiments were conducted in accordance with government policies and the Helsinki Declaration. The Ethics Committee of Huizhou Central People’s Hospital approved the study. **Supplementary Table 1** presents detailed clinical and pathological information on the patients. The methods used in this study are described in the section of Materials and Methods.

### Tertiary lymphoid structures evalution

Tertiary lymphoid structures (TLS) were both qualified and quantified using Hematoxylin and Eosin (H&E) staining or immunofluorescence, as detailed in prior studies (12, 13), with the number of TLS per square millimeter area being the specific measure utilized. For H&E-stained evaluation, A TLS was considered as germinal centers positive (mature TLS) if the TLS showed the characteristic morphology of proliferating centroblasts. For Immunofluorescence evaluation, TLS were identified as lymphocyte aggregates with histological features that included B cells (CD20), T cells (CD3), follicular dendritic cells (CD21 and CD23) in the intratumor, peritumor, and para-tumor areas. TLS was evaluated for its level of maturation in GC tissue. E-TLS, PFL-TLS, and SFL-TLS. These types differed in the presence of follicular dendritic cells (FDC) and their markers. Three types of TLS were identified: E-TLS, which are early TLS, consist of aggregates of CD20+ B cells without FDC. PFL-TLS, which are primary follicle-like TLS, have differentiated CD21+ FDC. SFL-TLS, which are secondary follicle-like TLS, have a germinal center that is notably visible through the presence of CD21+ CD23+ FDC.

### Calculation of tertiary lymphoid structure density

The determination of TLS density at intratumoral, peritumoral, and para-tumoral sites in breast cancer involved using the microscope with an eyepiece field of view of 22, counting TLS per 10X field. The calculation involved a diameter (d) of 0.22 mm, leading to an S of π/4*d^2 (3.8 mm^2). Following this, TLS density was calculated throughout the whole slide, as has been described in previously studies (14, 15).

### Immunofluorescence evaluation

Sections of 4-μm thickness were prepared from paraffin-embedded human breast cancer tissues for immunofluorescence staining, employing antibodies against human CD20 (1:500, Abcam, Cat# ab78237), CD3 (1:100, Abcam, ab5690. Cat# ab5690), CD21 (1:200, Abcam, Cat# ab75985), CD23 (1:400, Abcam, Cat# ab135386), CD8 (1:150, Abcam, Cat# ab237710), CD103 (1:500, Abcam, Cat# ab129202), Granzyme B (1:200, Abcam, Cat# ab255598), IFN-γ (1:50, proteintech, Cat# 15365-1-AP), CD56 (1:600, Abcam, Cat# ab220360), IgG (1:200, Abcam, Cat# ab181236), cleaved-caspase 3 (1:100, Abcam, Cat# Ab32042), CK-7 (1:1000, Abcam, Cat# ab181598), Ki-67 (1:600, Abcam, Cat# ab16667) followed by HRP-conjugated anti-rabbit IgG (ThermoFisher). For immunofluorescence staining, primary and secondary antibodies were incubated at 37°C for 2 hours. the sections staining was performed on an Alexa Fluor™ 488/555/647 Tyramide SuperBoost™ Kit (ThermoFisher). Positive cells were detected by confocal microscopy (Carl Zeiss LSM800). For cell counting in fluorescence-stained samples, we used QuPath, an open-source software designed for digital pathology image analysis. QuPath enabled us to perform automated cell counts across designated regions of interest, ensuring accuracy and reproducibility in the results. Specific fluorescent markers were optimized to minimize signal overlap, and cells were counted based on the presence of specific markers, ensuring precise identification and quantification across the designated regions of interest.

### The TCGA TCGA search protocol

Go to the TCGA homepage (tap me to enter) — Lunch Data Portal — Download Data — Data Matrix — Filter Settings: select Disease (BRCA - Breast Cancer), Data Type (RNA Seq), and Platform: genome-wide mRNA level (Illumina mRNA-seq).

### Statistical analysis

All data are presented as mean ± SEM. Non-compliance with parameter test conditions necessitated the use of the Mann-Whitney U test for statistical analyses, which were performed using two-tailed tests. The parameter test conditions used Welch’s ANOVA test. Kaplan–Meier overall survival and disease-free survival analyses of breast patients was performed using log rank. Correlation analyses were performed a Pearson, the two variables analyzed in these correlation analyses were normally distributed. Statistical analyses in the table I used a χ2 test. P values< 0.05 are statistically significant. The Cox proportional hazards regression model was utilized to compute HR and 95% CI, with statistical significance set at P < 0.05. Two or three independent experiments was performed in the data. Statistical analyses were executed with R (version 4.0.1), Caseviewer 2.2, QUPATH 0.4.3, and GraphPad Prism 8.0.

## Result

### Clinicopathological characteristics of patients with breast cancer

A total of 105 postoperative primary breast cancer patients were retrospectively recruited for the study, with their initial characteristics outlined in **Table 1**; **Supplementary Table 1**. All participants in the study were female, with a median diagnosis age of 50 years, ranging between 29 and 95 years. The number of tumors per patient ranged from 1 to 4, with the median being 1 tumor. Sizes of the tumors varied from 0.6 cm to 8 cm, with a median measurement of 2.5 cm. There were 76 tumors at stages I-II and 29 at stages IIIA-IIIC. In terms of menopausal status, there were 53 patients who were premenopausal and 52 who were postmenopausal.

**Table 1:** Associations between clinical factors in patients with breast cancer.

Characteristics	Low-mTLS (N=51)	High-mTLS (N=54)	Total (N=105)	*P*-value	FDR
TNM stages				1	1
I-II	37 (35.24%)	39 (37.14%)	76 (72.38%)		
IIIA-IIIC	14 (13.33%)	15 (14.29%)	29 (27.62%)		
Age (median, 50)				**0.02**	0.24
<50	31 (29.52%)	19 (18.10%)	50 (47.62%)		
>=50	20 (19.05%)	35 (33.33%)	55 (52.38%)		
menopause				**0.02**	0.37
Before	32 (30.48%)	21 (20.00%)	53 (50.48%)		
After	19 (18.10%)	33 (31.43%)	52 (49.52%)		
pathological type				1	1
breast ductal carcinoma	20 (19.05%)	21 (20.00%)	41 (39.05%)		
invasive breast cancer	31 (29.52%)	33 (31.43%)	64 (60.95%)		
Tumor number				0.2	1
1=<1	21 (20.00%)	30 (28.57%)	51 (48.57%)		
>1	30 (28.57%)	24 (22.86%)	54 (51.43%)		
Size (cm)				0.38	1
<2.5	23 (21.90%)	30 (28.57%)	53 (50.48%)		
>=2.5	28 (26.67%)	24 (22.86%)	52 (49.52%)		
ER				0.06	0.8
positive	40 (38.10%)	32 (30.48%)	72 (68.57%)		
negative	11 (10.48%)	22 (20.95%)	33 (31.43%)		
PR				0.1	1
positive	38 (36.19%)	31 (29.52%)	69 (65.71%)		
negative	13 (12.38%)	23 (21.90%)	36 (34.29%)		
CerB2				0.53	1
positive	37 (35.24%)	43 (40.95%)	80 (76.19%)		
negative	14 (13.33%)	11 (10.48%)	25 (23.81%)		
Her2				0.44	1
positive	5 (20.00%)	2 (8.00%)	7 (28.00%)		
negative	8 (32.00%)	10 (40.00%)	18 (72.00%)		
Ki67 (Median=40%)				0.15	1
<40	28 (26.67%)	21 (20.00%)	49 (46.67%)		
>=40	23 (21.90%)	33 (31.43%)	56 (53.33%)		
sentinel node				1	1
negative	33 (31.43%)	35 (33.33%)	68 (64.76%)		
positive	18 (17.14%)	19 (18.10%)	37 (35.24%)		
axillary lymph nodes				0.67	1
negative	36 (34.29%)	35 (33.33%)	71 (67.62%)		
positive	15 (14.29%)	19 (18.10%)	34 (32.38%)		

Data were expressed as n (%) and median. TLS, Mature tertiary lymphoid structures. Statistical analyses the table used a χ2 test. *P* values in bold are statistically significant.

### Identification of tertiary lymphoid structure

**Figure 1:**
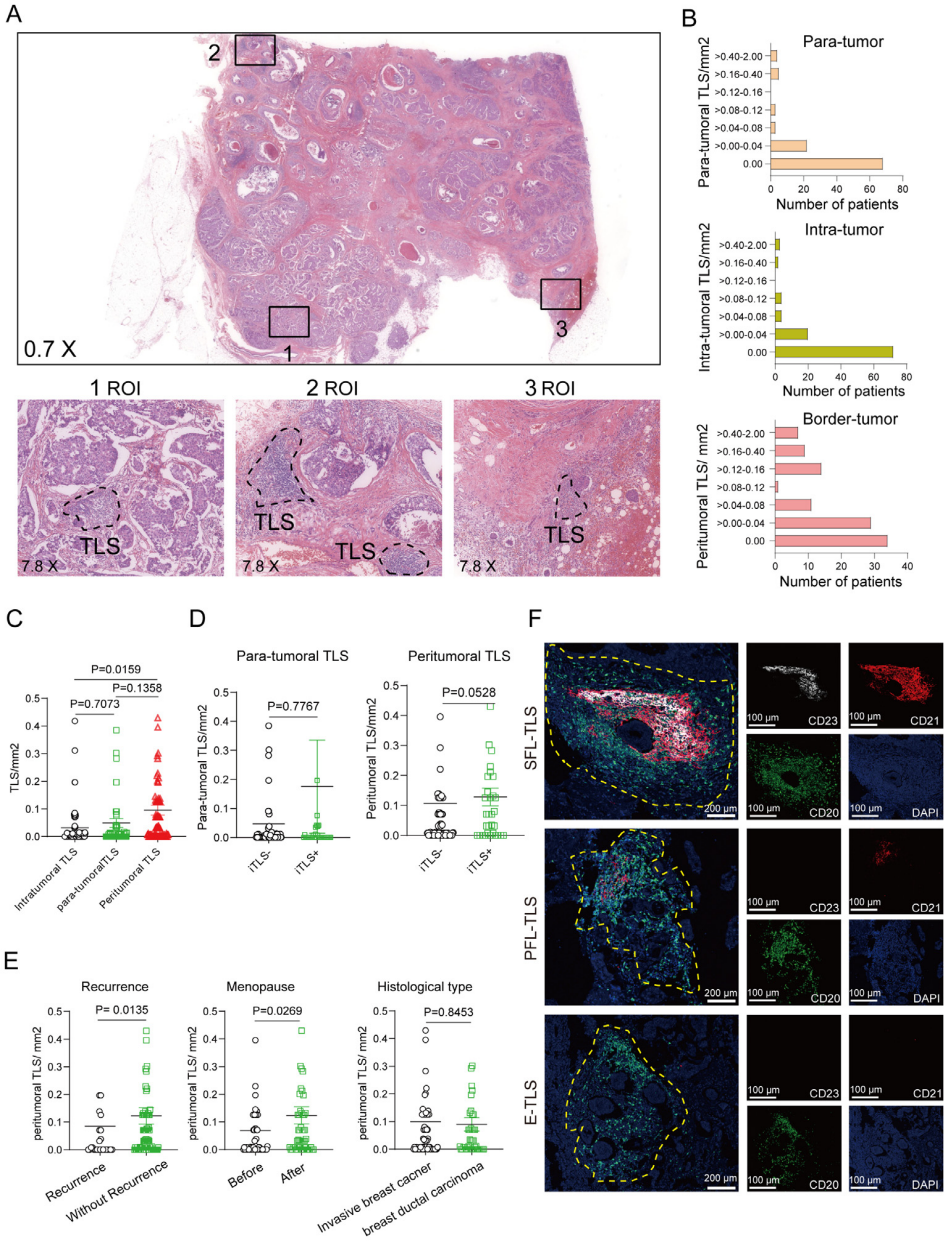
Detection and identification of tertiary lymphoid structures. Characterization of breast cancer-associated TLS. **(A)** Representative images of H&E-stained breast cancer tissue showing 3 regions of interest (ROI) in tumors. **(B, C)** The distribution of TLS density in intratumor, para-tumor and peritumor of breast cancer tissues was evaluated throughout the whole slide. **(D)** The density of pTLS was compared between breast cancer patients present or absent with iTLS. **(E)** The relationship between recurrence, Menopause, and Histological type. **(F)** the co-stained immunofluorescence of CD20+, CD21+, and CD23+. Data are presented as means ± SEM. Significance was determined by Welch’s ANOVA **(C)** two-tailed Mann-Whitney U **(D, E)**.

The initial evaluation of the presence and localization of TLS involved the examination of H&E-stained sections obtained from 105 patients with breast cancer at Huizhou Central Hospital. TLSs presented in many solid tumors and aggregated lymphocytes were considered as the typical structure of TLSs. From our H&E-staining of breast cancer, we observed the presence of multi-location in which TLS was found within tumor tissue (intratumor TLS, iTLS) or peritumoral region (peritumoral, pTLS) or distant normal tissue (para-tumoral TLS, para-TLS) (**Figure 1A**). To evaluate the TLS density, we quantified the numbers of TLSs in 5 random microscope bright light fields, and TLS density was calculated as the number of TLS per mm^2^ in different regions of tumor tissue. The distribution of TLS density was presented in **Figure 1B**. Our data showed that most TLSs located in the peritumor compared with those in intra-tumor and para-tumor in most patients with breast cancer (**Figure 1C**). We also examined the presence of iTLS relative to pTLS density and found a higher pTLS density in cases presence of iTLS (**Figure 1D**). Notably, the tumor tissue of relapsed or premenopausal breast cancer patients exhibited a low pTLS density. Still, we did not find any difference in pTLS density between different histological type (**Figure 1E**). Additionally, we also performed immunofluorescence staining (IF), found that TLS with a characteristic morphology of germinal centers was a hallmark of secondary follicle-like TLSs (SFL-TLS), containing mature CD21+ CD23+ follicular dendritic cells (FDC). Primary follicle-like TLSs (PFL-TLS) contained mature CD21+ FDC and lacking germinal centers (CD23-). Early TLSs (E-TLS) showed early lymphocytic aggregate without any apparent FDC networks or germinal centers (**Figure 1F**). In summary, we evaluated the TLS densities in breast cancer patients, and found that pTLS was the highest density among iTLS, pTLS and para-TLS.

### TLS density predicts survival of breast cancer

**Figure 2:**
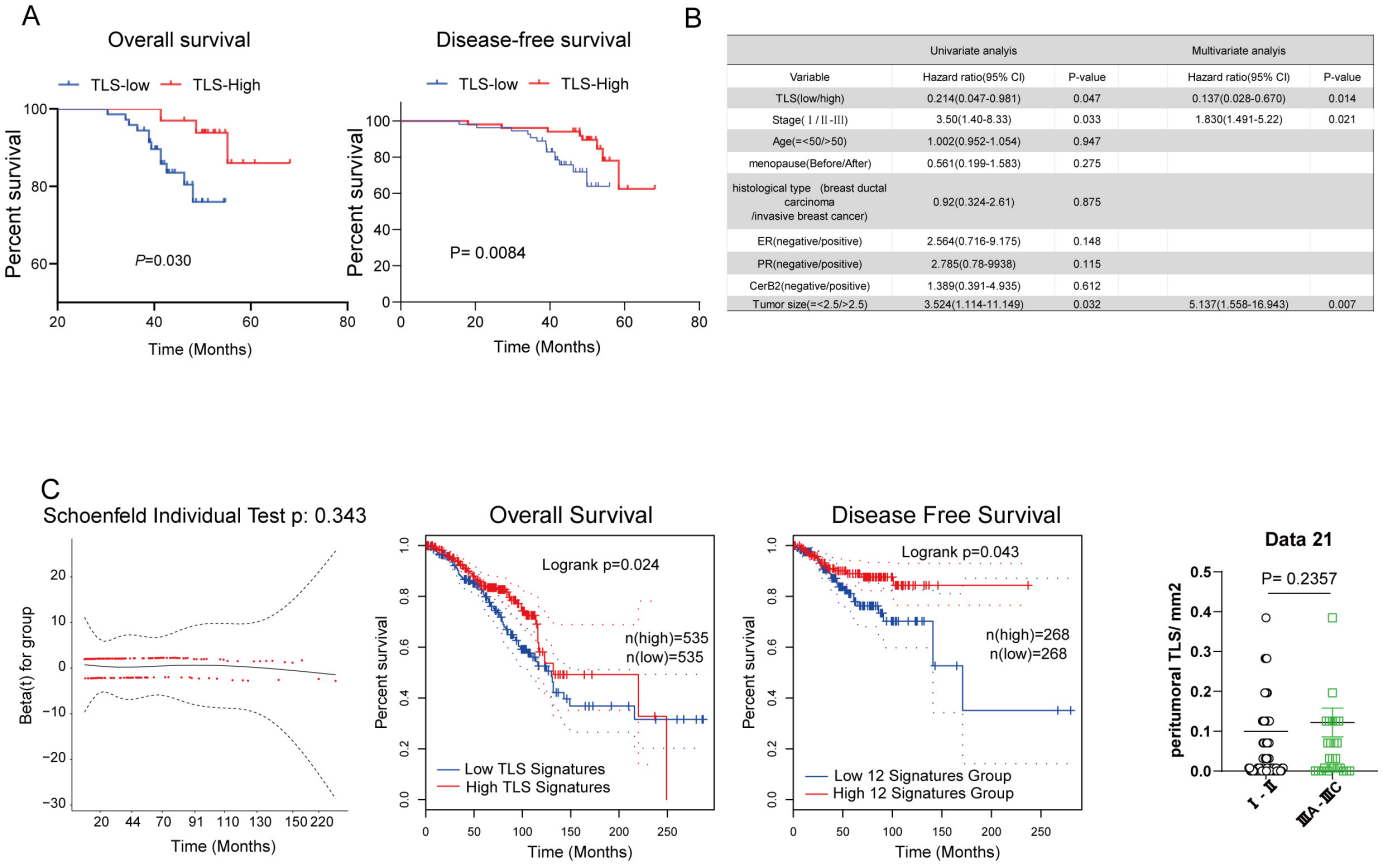
The correlation between tertiary lymphoid structures and prognostic significance of breast cancer patients. **(A)** Kaplan–Meier curve of TLS density determined by median of TLS density for Overall and Disease-free survival. **(B)** Hazard ratios and 95% CI in the clinical characterization of patients with breast cancer. **(C)** Kaplan–Meier overall survival and disease-free survival of breast cancer patients was performed based on the TLS signature in the BRCA TCGA dataset.

To investigate the prognostic effect of pTLS density on HCC patients, a threshold for separating patients with low and high pTLS densities was examined by using median of pTLS densities (**Figure 2A**). Kaplan-Meier survival analyses revealed that high pTLS density (> 0.0526 pTLS/mm2, n=54) significantly correlated with improved OS in 105 HCC patients from Huizhou Central Hospital (**Figure 2A**). Univariate analyses demonstrated a significant correlation between high pTLS density and longer OS (hazard ratio, 0.214; 95% CI, 0.047-0.981). When we grouped stage I separately from stages II-III, we observed a significant relationship with OS (**Figure 2B**). The findings were confirmed by the validation cohort of BRCA TCGA data set. In tumors with high pTLS densities, the presence of TLS still correlated with better Over Survival and Disease-Free Survival of breast cancer patients, suggesting its association with the best prognostic outcomes (**Figure 2C**). Taken together, these data indicate that pTLSs is a favorable prognostic factor for breast cancer patients.

### Peritumoral tertiary lymphoid structure was associated with CD103+CD8+ tissue-resident memory T cell infiltration

**Figure 3:**
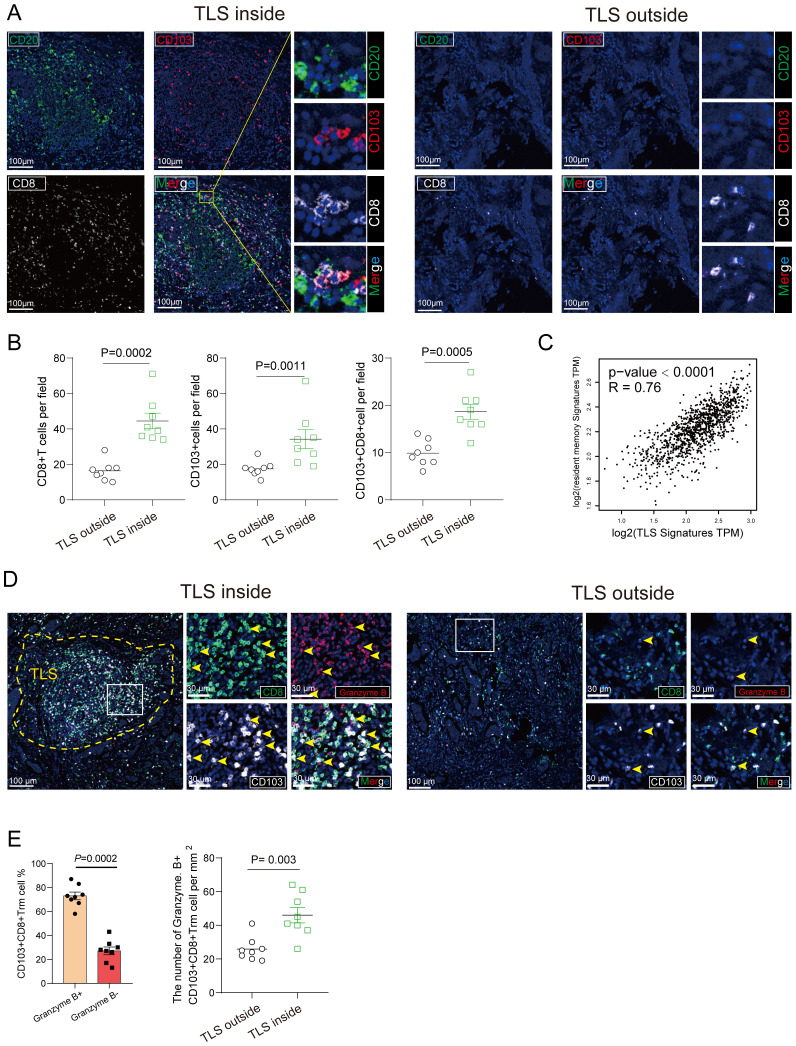
CD103+ CD8+ Trm cells distribution and its association with TLS. **(A)** The representative image of tumor section within mature TLS or TLS outside tissue from Breast cancer patients was stained by multiplex Immunofluorescence showing colocalization of CD103+ CD8+ Trm cells and CD20+ B cells. Scale bars, 100 μm. **(B)** The number of CD103+ cells, CD8+ T cell and CD103+ CD8+ Trm cells per mm2 was showed inside and outside the TLS area. **(C)** The correction between resident memory T cell signature and TLS signature. P values and R values were determined based on the analysis of Spearman’s correlation. **(D)** Multiplex Immunofluorescence of Granzyme **(B)** CD103+ CD8+ Trm cells were shown. The representative multi-immunofluorescence of breast cancer tissue was stained with CD8 (green), CD103 (gray) and Granzyme B (red). Scale bar, 100 μm. **(E)** The quantitation was shown. Data are presented as means ± SEM. Significance was determined by two-tailed Mann-Whitney U **(B)**.

**Figure 4:**
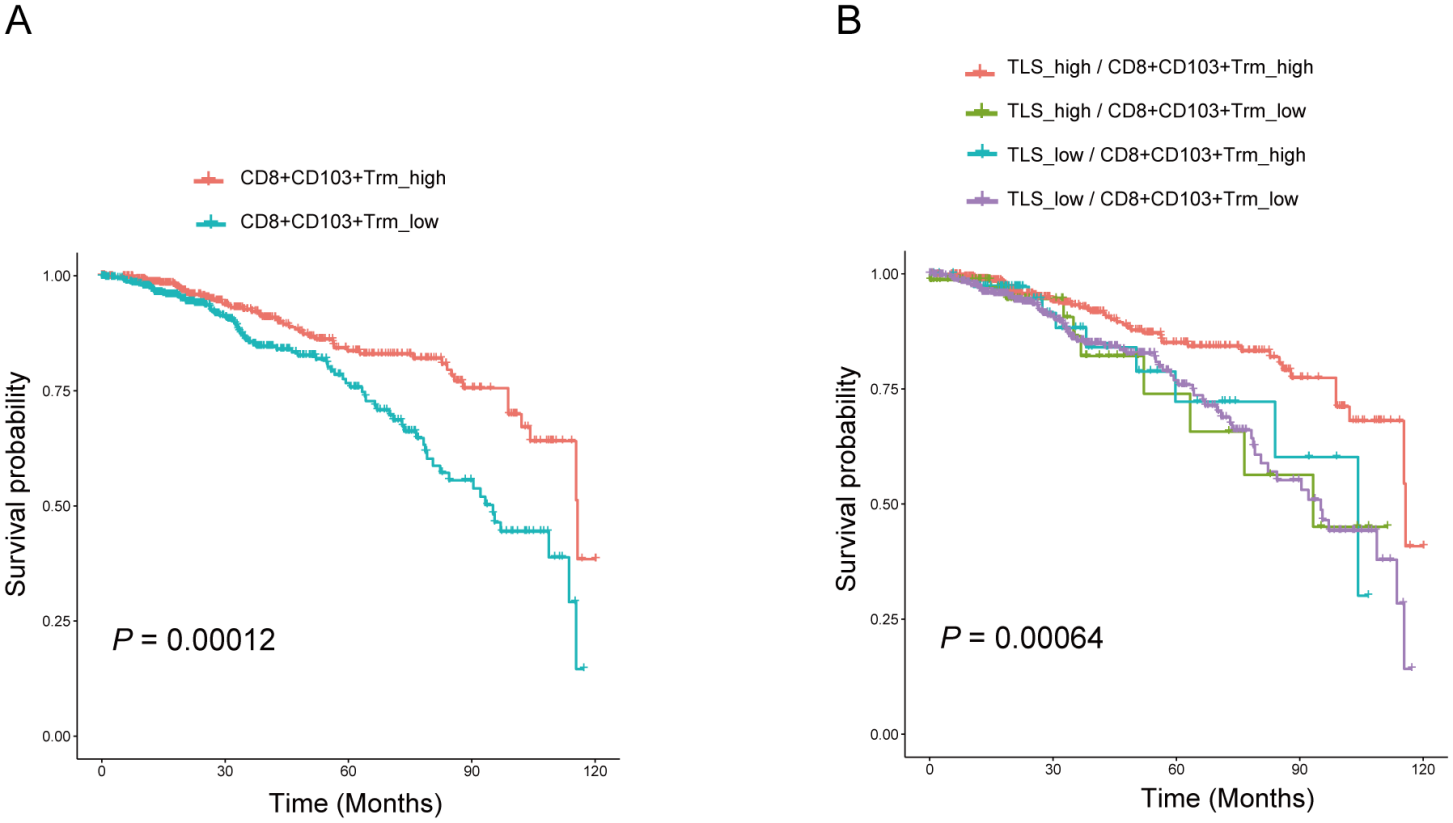
CD103+CD8+ Trm cells and TLS correlated with better prognosis in patients with breast cancer. **(A)** Kaplan–Meier overall survival and disease-free survival of breast cancer patients was performed based on the CD103+CD8+ Trm cells signature in the BRCA TCGA dataset. **(B)** Overall survival of patients with breast cancer based on CD103+CD8+ Trm cells signature and TLS signature according to the BRCA TCGA dataset. Analysis was performed using Kaplan-Meier.

Tumor-infiltrating lymphocytes (TILs) represent a more robust antigen-experienced, antitumor immune response, we hypothesized that TLS may support the activation of TIL attack against tumor cells. Immunohistochemistry using anti-CD103 antibody showed that CD103+ T cells were scattered around areas of lymphocyte aggregation as seen with anti-CD20 staining; these aggregates were considered TLSs (**Figure 3A**). Most CD8+ T cells had infiltrated not only into the intra-tumor, but also into the TLS of peritumor regions. Conversely, most CD103+ T cells were located within the border of TLS in peritumor regions and were considered tissue-resident memory T cells (**Figure 3A**). Immunofluorescence double staining for CD8 and CD103 showed that many CD103+ T cells express CD8 (**Figure 3A**). Interestingly, a significant increase in the percentage of CD103+ CD8+ T cluster was observed in the set of TLS-high group compared with TLS-low one (**Figure 3B**). These results indicated that CD103+ CD8+ Trm cells, one subtype of CD8+ T cells, infiltrated within TLS. It came to the same conclusion using the TCGA data set regarding breast cancer, tissue-resident memory T signature was related to TLS signature (**Figure 3C**). To examine the function of CD103+ CD8+ Trm cells, we analyzed the difference in cytokine production by CD103+ CD8+ Trm cells from 8 tumors. And we found that most Granzyme B+ CD103+ CD8+ Trm cells infiltrating within TLS rather than non-TLS one in breast cancer (**Figure 3D**). Additionally, we conducted a comparison of the number of GZMB+ CD8+ Trm cells within TLS versus TLS outside areas. This analysis confirmed the presence of significantly more effector (GZMB+) CD8+ Trm cells within mature TLS compared to TLS outside regions (**Figure 3E**), indicating a strong correlation between the effector function of CD103+ CD8+ Trm and TLS. Kaplan-Meier survival analyses revealed that high The CD103+ CD8+ Trm cells signature significantly correlated with improved prognosis in patients (**Figure 4A**). Furthermore, The Kaplan–Meier curves indicated that patients with high TLS signature and CD103+ CD8+ Trm cells signature in breast cancer conferred significantly best prognosis than those with low TLS signature and CD103+ CD8+ Trm cells signature (**Figure 4B**).

### Peritumoral tertiary lymphoid structure with multiple effector immune cells infiltration was associated enhancement of anti-tumor immunity in breast cancer

**Figure 5:**
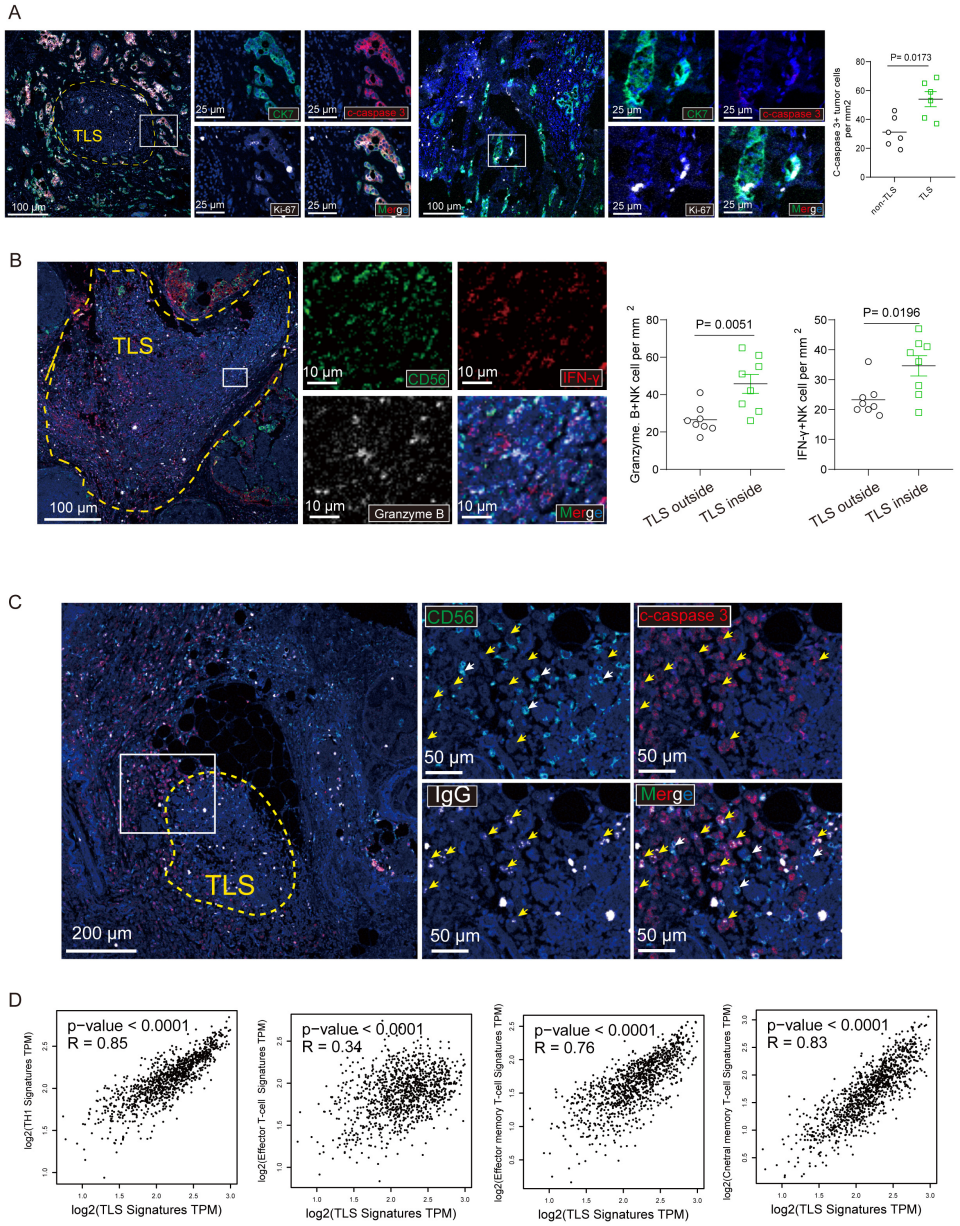
CD103+ CD8+ Trm cells and NK cells within mature TLS secreted effector molecules. **(A)** The representative immunostaining of breast cancer tissue with mature TLS was stained with CK7, cleaved caspase-3 (c- caspase-3) and Ki-67. Scale bar, 100 μm. the number of c- caspase-3 tumor cells per mm2 was shown. **(B)** The representative multi-immunofluorescence of breast cancer tissue with mature TLS was stained with CD56 (green), Granzyme B (gray) and IFN-γ (red). Scale bar, 100 μm. **(C)** The representative immunostaining of breast cancer tissue with TLS was stained with CD56, cleaved caspase-3 (c- caspase-3) and immunoglobulin G (IgG). Scale bar, 100 μm. **(D)** The correction between TLS signature and TH1 signature, Effector T-cell signature, Effector memory T-cell signature, central memory T-cell signature, respectively. P values and R values were determined based on the analysis of Spearman’s correlation.

To unveil potential functions of TLS of breast cancer, we searched for signs of proliferation and apoptosis occurring in tumor cells, identified by their typical large nucleus and CK7+ phenotype, using an antibody that detects Ki-67 and cleaved caspase 3. **Figure 5A** illustrates labeling of cleaved caspase 3 in a TLS+. The density of cleaved caspase 3+ tumor cells was significantly higher in tumors around TLS.

Recent study has previously revealed that presence of intratumoral TLS correlated with multiple tumor-infiltrating immune cells. We also examined the tumor-infiltrating immune cells in breast cancer tissues by immunofluorescent staining (**Figure 5B**). In addition, we determined the association between pTLS and tumor-infiltrating immune cells. pTLS-high tumors were significantly associated with increased CD56+ NK cells which secreted effector molecule Granzyme B and IFN-γ (**Figure 5B**). As Of note, a typical large nucleus tumor by conjugated anti-human IgG was observed as illustrated in a TLS+ tumor (**Figure 5C**). We addressed the question of the mechanisms that may be at work in inducing apoptosis of tumor cells. Since NK cells are the main effectors of antibody-dependent cellular cytotoxicity (ADCC), we evaluated the overall density of CD56+ NK cells on breast cancer tissues and found that tumors with both high numbers of apoptotic cells and a high percentages of IgG-stained tumor cells were more infiltrated by CD56+ NK than tumors with high numbers of apoptotic cells but low percentages of IgG-stained tumor cells (**Figure 5C**). Additionally, using the TCGA data set regarding breast cancer, we also found that TH1, effector T cell, effector memory T cell and central memory T cell signature were positively related to TLS signature (**Figure 5D**). Taken together, TLS with multiple effector immune cells infiltration was associated enhancement of anti-tumor immunity in breast cancer.

## Discussion

The current study delves into the relationship between tertiary lymphoid structures (TLS) and their associated immune cells in breast cancer, aiming to shed light on the mechanisms underlying their role in anti-tumor immunity. While prior research has acknowledged the importance of TLS in various solid tumors and their correlation with better prognoses, the precise mechanisms remained elusive (9, 16). Firstly, unlike previous studies that primarily focused on the presence or absence of TLS, our research delves deeper by assessing the maturity of TLS and its correlation with key immune cells, particularly CD103+ CD8+ Trm and NK cells. Secondly, our study not only confirms the favorable prognosis associated with the presence of TLS in breast cancer patients but also introduces potential additional prognostic markers—CD103+ CD8+ Trm cells and NK cells within TLS. Finally, by demonstrating the enhanced effector function of CD103+ CD8+ Trm cells and NK cells within mature TLS, we establish a strong correlation between immune cell functionality and TLS, offering new insights into their collective role in anti-tumor immunity.

To the best of our knowledge, this study is the first large comprehensive report on the importance of CD103+ CD8+ Trm cell and TLS in breast cancer. We found that TLSs are mainly present in peritumoral carcinoma *in situ* in breast cancer. Although there is no standardized classification of TLS maturity, we used the classification method to classify the TLSs into three mature stages, including E-TLSs, PFL-TLSs, and SFL-TLSs. We found that TLS with a characteristic morphology of germinal centers was a hallmark of secondary follicle-like TLSs (SFL-TLS), containing mature CD21+ CD23+ follicular dendritic cells (FDC). Primary follicle-like TLS (PFL-TLS) contained mature CD21+ FDC and lacking germinal centers (CD23-). Early TLS (E-TLS) showed early lymphocytic aggregate without any apparent FDC networks or germinal centers. For the first time, we divided patients into two levels based on the mature state of TLS; 1) mature TLS: only E-TLS or no TLS with no PFL-TLS and SFL-TLS; 2) immature TLS: PFL-TLS and SFL-TLS in the tumor, and without E-TLSs. The results showed that patients in mature TLS had the best over survival than that in immature TLS. This was consistent with the findings in primary pancreatic adenocarcinoma, colorectal cancer and lung squamous cell carcinoma that found that patients with GC reaction had a better progression-free survival, and overall survival (15–17). This indicates that mature TLS play pivotal roles in the anti-tumor immune response.

The recent successful treatment of immunotherapy in various solid and hematological tumors suggests that immunotherapy has a better antitumor effect, and cytotoxic CD8+ lymphocytes infiltrated in tumors have been widely studied and associated with improved survival in most patients (18, 19). Many studies have now found that there are also lymphocyte aggregates in the tumor microenvironment, which resemble secondary lymphoid structures called tertiary lymphoid structures (TLS); the presence of tertiary lymphoid structures has been closely associated with a good prognosis for patients in a variety of tumors (9, 10, 20). Our study also found that TLS in breast cancer can be associated with good prognosis. Prolonged exposure to inflammatory infiltration and modulation by cytokines and chemokines can promote the formation of tertiary lymphoid structures in peripheral tissues, and a large number of studies have found tertiary lymph node formation in tissues with autoimmune diseases, transplant rejection and chronic diseases (21). The vast majority of tumor-associated TLS have antitumor functions and are indicators of a good tumor prognosis. Several studies have pointed out the infiltration of TLS defined by HEV, CD4 or TFH cells, B-cells and mature DCs TH1 and cytotoxic T-cells are associated and promote antitumor effects (9). Numerous studies have confirmed the better antitumor effect of tumor infiltrating CD8+ T cells, for this reason, researchers found an independent antitumor function of TLS by excluding the role of CD8+ T cells through multifactorial analysis (10). In TLS with low DC infiltration, it has a poor prognostic effect even though it contains a large number of CD8+ T cells (22, 23). Interestingly, we found that TLS in breast cancer was able to correlate with a good prognosis, which was closely associated with the density of CD103+CD8+ Trm cells and NK cell infiltration in TLS, and also found that CD103+CD8+ Trm cells and NK cells secreted a large number of effector molecules such as IFN-γ and Granzyme B. Suggesting that the CD103+ CD8+Trm cells and NK cells within TLS favor anti-tumor immune responses.

Although the exact mechanism by which tissue-resident memory T cells are preferentially localized within TLS remains to be fully elucidated, it has been reported that CXCL13 plays a pivotal role as the primary molecular driver behind the formation of TLS in the tumor microenvironment (TME) (15, 24, 25). Activated CD103+ cytotoxic T lymphocytes (CTLs) play a crucial role in the recruitment of B cells to the tumor microenvironment through the secretion of CXCL13. Tumors characterized by a high mutation load and rich in CD8+ T cells exhibit elevated levels of CXCL13 and CD103, correlating with significantly increased B cell presence across various tumor types. This enhanced B cell infiltration is particularly pronounced in tumors with abundant TLS, suggesting a vital role for activated CD103+ CTLs in mediating this process (25). A prior study examining the distribution of CD8+CD103+ Trm cells in gastric carcinoma revealed similar outcomes. CD103+ T cells were predominantly located around TLSs, and patients exhibiting a high expression of CD103 (CD103 High) displayed an increased number of TLSs (33735485). Furthermore, tumor infiltrating CD103+Trm cell was prognostic factor for gastric cancer patients (26). However, this study mainly focused on the relationship between TLS maturity and CD103+ CD8+ Trm in breast cancer. Importantly, CD103+ CD8+ Trm cells in TLSs served as an independent prognosticator of breast cancer. Additionally, TLS and CD103+ CD8+ Trm cells located within TLS were linked to enhanced overall survival rates in breast cancer patients. Immune cells are essential components of tumor environment, reflecting the immunogenicity of tumor, and they are important biomarkers for immunotherapy for patients with breast cancer (27, 28). It has been reported that in invasive breast cancer there is no difference in the distribution of immune cells between the intratumor and peritumor, 32 but in our study, peritumoral immune cells were more abundant than intratumor in breast cancer. Specifically, we found that CD103+ CD8+ Trm cells and NK cells were more in the peritumor than in the peritumor. Previously studies have shown a decreasing trend of TILs in metastatic breast cancer compared to primary breast cancer (29, 30). Interestingly, we found that TLS in breast cancer was able to correlate with good prognosis, and the density of CD103+ CD8+ Trm cells and NK cell infiltration in TLS were closely correlated. The combination of TLS with CD103+ CD8+ Trm cells predicted a better good prognosis. This study investigated the relationship between infiltration of different immune subpopulations of TLS at different areas in breast cancer and patient prognosis. Future studies are still needed to determine the role of immune cells in breast cancer.

In conclusion, our data highlight that the proportion of CD103+ CD8+ Trm cells within pTLSs was significantly increased with the maturation of TLSs. TLS with multiple effector immune cell infiltration was associated with enhanced anti-tumor immunity in breast cancer. These results indicate a close relationship between CD103+ CD8+ Trm cell and pTLS maturity, suggesting a strong correlation between the effector function of CD103+ CD8+ Trm and NK cells and the maturity of pTLSs. Furthermore, patients with a combination feature of TLS and CD103+ CD8+ Trm cells showed a good prognosis. The combination of pTLS maturity and CD103+ CD8+ Trm cells proportion could be used as a biomarker to predict the prognosis of breast cancer patients.

## Data Availability

The raw data supporting the conclusions of this article will be made available by the authors, without undue reservation.
