# Supplementary material for: Mature tertiary lymphoid structure associated CD103+ CD8+ Trm cells determined improved anti-tumor immune in breast cancer
Source: Front Oncol. 2025 Jan 24;15:1480461. doi: 10.3389/fonc.2025.1480461 (PMC11802804; doi:10.3389/fonc.2025.1480461)
Supplement: Supplementary file 1 [file Table1.docx]

Supplementary Table 1. 105 cases of Breast cancer in clinical characteristics.

| Patents | TNM Stage | | | | Pathological Type | Age | Tumor number | Tumor Size (cm) | ER (% or +) | PR (% or +) | CerB (-/ +/ ++ /+++) | her2 (+/-) | Ki67 (%) |
| --- | --- | --- | --- | --- | --- | --- | --- | --- | --- | --- | --- | --- | --- |
| P1 | T2 | N1 | M0 | ⅡB | Breast ductal carcinoma | 44 | 2 | 2.7 | +++ | +++ | ++ | - | 30 |
| P2 | T1 | N1 | M0 | ⅡA | Invasive breast cancer | 32 | 1 | 2 | 90 | 50 | ++ | - | 20 |
| P3 | T1 | N1 | M0 | ⅡA | Breast ductal carcinoma | 53 | 1 | 2 | - | 2 | +++ |  | 40 |
| P4 | T2 | N2 | M0 | ⅢA | Invasive breast cancer | 53 | 2 | 5 | - | - | +++ |  | 40 |
| P5 | T1 | N3 | M0 | Ⅲc | Breast ductal carcinoma | 59 | 1 | 1 | 90 | 30 | - |  | 40 |
| P6 | T2 | N1 | M0 | ⅡB | Invasive breast cancer | 40 | 2 | 4 | 90 | 90 | ++ |  | 40 |
| P7 | T2 | N3 | M0 | ⅢC | Breast ductal carcinoma | 76 | 2 | 5 | 100 | 60 | ++ | - | 20 |
| P8 | T1 | N0 | M0 | Ⅰ | Breast ductal carcinoma | 44 | 1 | 2 | - | - | - |  | 70 |
| P9 | T1 | N1 | N0 | ⅡA | Invasive breast cancer | 47 | 1 | 1.8 | 90 | 90 | +++ |  | 40 |
| P10 | T1 | N0 | M0 | ⅡA | Invasive breast cancer | 49 | 2 | 2.5 | 90 | 90 | ++ |  | 10 |
| P11 | T4 | N0 | M0 | IIIB | Invasive breast cancer | 57 | 4 | 7.5 | - | - | + |  | 40 |
| P12 | T1 | N2 | M0 | IIIA | Invasive breast cancer | 63 | 1 | 2.5 | - | - | ++ |  | 30 |
| P13 | T2 | N0 | M0 | ⅡA | Breast ductal carcinoma | 56 | 2 | 4.5 | - | - | +++ |  | 30 |
| P14 | T1 | N0 | M0 | Ⅰ | Breast ductal carcinoma | 49 | 1 | 2 | 90 | 90 | ++ |  | 20 |
| P15 | T1 | N3 | M0 | ⅢC | Invasive breast cancer | 61 | 1 | 1.7 | 90 | 70 | ++ |  | 30 |
| P16 | T1 | cN0 | M0 | Ⅱ | Invasive breast cancer | 54 | 1 | 1.2 | 90 | 80 | + |  | 30 |
| P17 | T1 | CM0 | M0 | Ⅰ | Breast ductal carcinoma | 47 | 1 | 1.2 | 90 | 90 | ++ | - | 20 |
| P18 | T1 | cN1 | M0 | ⅡA | Breast ductal carcinoma | 54 | 1 | 2 | 90 | 5 | + |  | 40 |
| P19 | T1 | cN0 | M0 | I | Breast ductal carcinoma | 58 | 1 | 1.5 | - | - | +++ |  | 30 |
| P20 | T2 | N0 | M0 | ⅡA | Invasive breast cancer | 44 | 2 | 2.5 | 80 | 80 | ++ | - | 20 |
| P21 | T2 | N1 | M0 | ⅡB | Breast ductal carcinoma | 56 | 2 | 4 | 80 | 40 | ++ | + | 30 |
| P22 | T2 | N1 | M0 | IIB | Invasive breast cancer | 43 | 2 | 2.2 | 90 | 90 | - |  | 30 |
| P23 | T2 | N0 | M0 | Ⅱ | Invasive breast cancer | 48 | 2 | 4.3 | 60 | 40 | +++ |  | 40 |
| P24 | T2 | N0 | M0 | ⅡA | Invasive breast cancer | 59 | 2 | 2.8 | - | - | - |  | 60 |
| P25 | T1 | N1 | M0 | ⅡA | Breast ductal carcinoma | 46 | 2 | 3 | - | - | + |  | 70 |
| P26 | T1 | N0 | M0 | Ⅰ | Invasive breast cancer | 52 | 1 | 2 | - | - | +++ |  | 40 |
| P27 | T1 | N1 | M0 | ⅡA | Breast ductal carcinoma | 63 | 1 | 1.5 | - | - | - |  | 50 |
| P28 | T1 | N1 | M0 | ⅡA | Invasive breast cancer | 47 | 1 | 2 | 70 | 60 | ++ | - | 40 |
| P29 | T2 | N1 | M0 | ⅡB | Breast ductal carcinoma | 51 | 2 | 3.5 | 80 | 60 | ++ | - | 30 |
| P30 | T1 | N0 | M0 | I | Invasive breast cancer | 52 | 1 | 3 | 90 | 90 | ++ | - | 5 |
| P31 | T2 | N0 | M0 | IIA | Invasive breast cancer | 47 | 2 | 3 | 80 | 5 | ++ | - | 30 |
| P32 | T1 | cN0 | M0 | Ⅰ | Breast ductal carcinoma | 54 | 1 | 1.5 | 60 | 50 | - |  | 15 |
| P33 | T1 | N1 | M0 | ⅡA | Invasive breast cancer | 50 | 1 | 1.2 | - | - | - |  | 80 |
| P34 | T1 | N3 | M0 | IIIC | Invasive breast cancer | 61 | 1 | 1.5 | - | - | +++ |  | 40 |
| P35 | T1 | N0 | M0 | I | Invasive breast cancer | 53 | 2 | 2.8 | 90 | 80 | ++ | + | 60 |
| P36 | T2 | N0 | M0 | ⅡA | Breast ductal carcinoma | 46 | 2 | 2.2 | 90 | 90 | + |  | 30 |
| P37 | T2 | N2 | M0 | ⅢA | Breast ductal carcinoma | 65 | 2 | 4 | - | - | - |  | 60 |
| P38 | T1 | N0 | M0 | I | Invasive breast cancer | 56 | 1 | 1 | 100 | 30 | ++ | - | 40 |
| P39 | T1 | N1 | M0 | ⅡA | Invasive breast cancer | 48 | 1 | 1.5 | 90 | 80 | ++ |  | 20 |
| P40 | T1 | N1 | M0 | IIA | Breast ductal carcinoma | 51 | 1 | 1.5 | - | - | ++ | + | 40 |
| P41 | T2 | N1 | M0 | ⅡB | Breast ductal carcinoma | 68 | 2 | 5 | - | - | + |  | 80 |
| P42 | T2 | N3 | M0 | ⅢC | Invasive breast cancer | 46 | 2 | 4 | - | - | +++ |  | 60 |
| P43 | T1 | N0 | M0 | I | Breast ductal carcinoma | 43 | 1 | 1 | 70 | 70 | + |  | 40 |
| P44 | T2 | N2 | M0 | ⅢA | Breast ductal carcinoma | 80 | 2 | 3.5 | - | - | + |  | 80 |
| P45 | T1 | cN0 | M0 | Ⅰ | Invasive breast cancer | 46 | 1 | 1 | 80 | 60 | ++ | + | 10 |
| P46 | T2 | N2 | M0 | IIIA | Breast ductal carcinoma | 52 | 2 | 2.5 | - | - | ++ | - | 80 |
| P47 | T1 | N1 | M0 | IIA | Breast ductal carcinoma | 75 | 1 | 2 | 90 | - | ++ | + | 4 |
| P48 | T2 | N1 | M0 | ⅡB | Breast ductal carcinoma | 49 | 2 | 3.5 | 80 | 80 | + |  | 10 |
| P49 | T1 | N1 | M0 | ⅡA | Breast ductal carcinoma | 36 | 1 | 2 | - | - | +++ |  | 50 |
| P50 | T2 | N1 | M0 | ⅡB | Breast ductal carcinoma | 34 | 2 | 4 | - | 5 | - |  | 80 |
| P51 | T2 | N0 | M0 | ⅡA | Breast ductal carcinoma | 40 | 2 | 2.5 | 90 | 80 | ++ |  | 20 |
| P52 | T1 | bN0 | M0 | Ⅰ | Breast ductal carcinoma | 55 | 1 | 1 | 90 | 10 | - |  | 40 |
| P53 | T2 | N2 | M0 | ⅢA | Invasive breast cancer | 51 | 2 | 4 | - | - | +++ |  | 40 |
| P54 | T2 | N1 | M0 | ⅡA | Breast ductal carcinoma | 69 | 2 | 3 | 90 | 50 | ++ |  | 20 |
| P55 | T1 | N0 | M0 | ⅠA | Breast ductal carcinoma | 42 | 1 | 1.5 | 90 | 90 | + |  | 20 |
| P56 | T3 | N3 | M0 | IIIC | Breast ductal carcinoma | 41 | 3 | 6 | 70 | 30 | +++ |  | 40 |
| P57 | T1 | cN1 | M0 | Ⅱ | Breast ductal carcinoma | 43 | 1 | 1.5 | 60 | 60 | ++ |  | 15 |
| P58 | T1 | N1 | M0 | ⅡA | Breast ductal carcinoma | 43 | 1 | 1.6 | 70 | 70 | ++ | + | 40 |
| P59 | T2 | N3 | M0 | ⅢC | Breast ductal carcinoma | 63 | 2 | 4 | 90 | 40 | - |  | 40 |
| P60 | T2 | Ns | n0 | M0 ⅡA | Breast ductal carcinoma | 56 | 2 | 2.5 | 90 | - | - |  | 40 |
| P61 | T2 | N0 | M0 | ⅡA | Breast ductal carcinoma | 47 | 2 | 2.6 | 90 | 90 | + |  | 30 |
| P62 | T2 | N0 | M0 | ⅡA | Breast ductal carcinoma | 29 | 2 | 3 | 80 | 90 | ++ |  | 50 |
| P63 | T1 | N0 | M0 | I | Invasive breast cancer | 42 | 1 | 1.3 | 90 | 90 | ++ | - | 40 |
| P64 | T2 | N1 | M0 | IIB | Breast ductal carcinoma | 68 | 2 | 5 | - | - | - |  | 90 |
| P65 | T2 | N0 | M0 | ⅡA | Breast ductal carcinoma | 67 | 2 | 2.5 | 10 | 20 | ++ | - | 40 |
| P66 | T2 | N1 | M0 | IIA | Breast ductal carcinoma | 48 | 1 | 2 | 60 | 20 | - |  | 30 |
| P67 | T2 | N1 | M0 | ⅡB | Invasive breast cancer | 34 | 2 | 2.5 | 90 | 80 | - |  | 40 |
| P68 | T2 | N0 | M0 | ⅡA | Breast ductal carcinoma | 43 | 2 | 3 | 90 | 90 | ++ | - | 20 |
| P69 | T2 | N1 | M0 | ⅡB | Invasive breast cancer | 45 | 2 | 3.5 | 80 | 80 | + |  | 20 |
| P70 | T1 | N0 | M0 | I | Invasive breast cancer | 61 | 1 | 1.3 | - | - | + |  | 40 |
| P71 | T2 | N2 | M0 | ⅢA | Invasive breast cancer | 95 | 1 | 2 | 100 | 30 | + |  | 30 |
| P72 | T1 | bN | 0M | 0 Ⅰ | Invasive breast cancer | 50 | 1 | 0.6 | 90 | 90 | ++ | + | 20 |
| P73 | T1 | cN | 0M | 0 Ⅰ | Breast ductal carcinoma | 43 | 1 | 1.5 | 70 | 80 | ++ | - | 20 |
| P74 | T2 | N2 | M0 | IIIa | Invasive breast cancer | 49 | 2 | 3 | - | - | +++ |  | 50 |
| P75 | T2 | N0 | M0 | ⅡA | Breast ductal carcinoma | 61 | 2 | 2.8 | - | - | - |  | 40 |
| P76 | T1 | N0 | M0 | I | Breast ductal carcinoma | 54 | 1 | 1.3 | 20 | - | ++ | - | 50 |
| P77 | T2 | N2 | M0 | IIIA | Invasive breast cancer | 46 | 2 | 2.5 | 50 | 40 | +++ |  | 25 |
| P78 | T1 | N3 | M0 | ⅢC | Breast ductal carcinoma | 49 | 1 | 2 | 80 | 30 | ++ | - | 20 |
| P79 | T1 | bN0 | M0 | Ⅰ | Invasive breast cancer | 54 | 1 | 0.8 | 90 | 10 | ++ |  | 30 |
| P80 | T2 | N2 | N0 | IIIA | Breast ductal carcinoma | 54 | 2 | 3.5 | 90 | 70 | + |  | 25 |
| P81 | T1 | N0 | M | I | Breast ductal carcinoma | 47 | 1 | 1.2 | 60 | 20 | +++ |  | 30 |
| P82 | T2 | N2 | M0 | ⅢA | Breast ductal carcinoma | 54 | 2 | 3.5 | 80 | 1 | - |  | 30 |
| P83 | T2 | N0 | M0 | ⅡA | Breast ductal carcinoma | 51 | 2 | 2.6 | - | - | - |  | 60 |
| P84 | T2 | N3 | M0 | IIIC | Breast ductal carcinoma | 50 | 2 | 3 | - | - | +++ |  | 50 |
| P85 | T2 | N3 | M0 | ⅢC | Invasive breast cancer | 43 | 2 | 3 | 80 | 10 | ++ |  | 40 |
| P86 | T2 | N1 | M0 | ⅡB | Breast ductal carcinoma | 44 | 2 | 2.8 | 70 | 40 | ++ |  | 40 |
| P87 | T1 | cN0 | M0 | Ⅰ | Breast ductal carcinoma | 48 | 1 | 2 | - | - | +++ |  | 80 |
| P88 | T1 | cN0 | M0 | Ⅰ | Breast ductal carcinoma | 61 | 2 | 2.5 | 90 | - | - |  | 15 |
| P89 | T2 | N2 | M0 | IIIA | Invasive breast cancer | 47 | 2 | 2.3 | 80 | 90 | ++ |  | 80 |
| P90 | T1 | N0 | M0 | ⅢC | Breast ductal carcinoma | 47 | 1 | 2 | 95 | 60 | ++ |  | 30 |
| P91 | T1 | N0 | M0 | Ⅰ | Breast ductal carcinoma | 60 | 1 | 0.8 | 90 | 10 | + |  | 20 |
| P92 | T1 | N0 | M0 | Ⅰ | Breast ductal carcinoma | 31 | 1 | 1 | 90 | 80 | - |  | 10 |
| P93 | T1 | cN0 | M0 | 0 Ⅰ | Breast ductal carcinoma | 49 | 1 | 1.8 | 90 | 70 | +++ |  | 40 |
| P94 | T1 | N3 | M0 | ⅢC | Invasive breast cancer | 58 | 1 | 2 | - | - | ++ | - | 20 |
| P95 | T1 | cN0 | M0 | Ⅰ | Invasive breast cancer | 49 | 1 | 1.2 | 90 | 20 | + |  | 30 |
| P96 | T1 | N0 | M0 | Ⅰ | Invasive breast cancer | 60 | 1 | 2 | 90 | 60 | +++ |  | 30 |
| P97 | T3 | N2 | M0 | ⅢA | Breast ductal carcinoma | 62 | 3 | 8 | 90 | - | - |  | 30 |
| P98 | T2 | N0 | M0 | ⅡA | Breast ductal carcinoma | 57 | 2 | 4 | 90 | 10 | - |  | 30 |
| P99 | T1 | N1 | M0 | IIA | Breast ductal carcinoma | 47 | 1 | 2 | 80 | 80 | - |  | 40 |
| P100 | T1 | cN0 | M0 | Ⅰ | Breast ductal carcinoma | 48 | 1 | 1.2 | - | - | - |  | 80 |
| P101 | T2 | N2 | M0 | ⅢA | Breast ductal carcinoma | 55 | 2 | 5 | 80 | 30 | + |  | 30 |
| P102 | T2 | N2 | M0 | ⅢA | Breast ductal carcinoma | 62 | 2 | 3.5 | 90 | 80 | + |  | 30 |
| P103 | T2 | N2 | M0 | ⅢA | Breast ductal carcinoma | 67 | 2 | 4 | 90 | 70 | - |  | 50 |
| P104 | T1 | CN0 | M0 | Ⅰ | Breast ductal carcinoma | 58 | 1 | 1.8 | - | - | +++ |  | 60 |
| P105 | T2 | N1 | M0 | ⅡB | Invasive breast cancer | 41 | 2 | 4 | - | - | +++ |  | 60 |
